# Associations of dietary indices with biomarkers of dietary exposure and cardiovascular status among adolescents in Germany

**DOI:** 10.1186/1743-7075-9-92

**Published:** 2012-10-24

**Authors:** Julia Truthmann, Almut Richter, Silke Thiele, Larissa Drescher, Jutta Roosen, Gert BM Mensink

**Affiliations:** 1 Robert Koch Institute Berlin, Department of Epidemiology and Health Reporting, Postbox 65 02 61, D-13302, Berlin, Germany; 2 Christian-Albrechts-University of Kiel, Department of Food Economics and Consumption Studies, Olshausenstraße 40, D-24098, Kiel, Germany; 3 TU München, School of Management, Marketing and Consumer Research, Alte Akademie 16, D-85350, Freising, Germany

**Keywords:** Dietary indices, Diet quality, Cardiovascular status, Nutritional epidemiology, Adolescents, Germany

## Abstract

**Background:**

Adolescence is an important life stage for the development of dietary preferences and health behaviour. Longitudinal studies indicated that cardiovascular status in adolescence predicts cardiovascular risk marker values in adulthood. Several diet quality indices for adolescents have been developed in the past, but literature concerning associations between indices and biomarkers of dietary exposure and cardiovascular status is rather sparse. Hence, the aim of this study was to analyse associations of dietary indices with biomarkers of dietary exposure and cardiovascular status.

**Methods:**

For the present analysis, data from the German Health Interview and Examination Survey for Children and Adolescents (*KiGGS* 2003–2006) were used. The analysis included 5,198 adolescents, aged 12 to 17 years. The Healthy Food Diversity Index (*HFD*), the Healthy Nutrition Score for Kids and Youth (*HuSKY*), the Indicator Food Index (*IFI*) and a simple fruit/vegetable intake index were derived from food frequency questionnaire information to indicate a healthy diet. Adjusted mean values for homocysteine, uric acid, *CRP*, total cholesterol, *HDL-C*, ferritin, *HbA1c*, folate, vitamin B_12_ and *BMI* were calculated using complex-samples general linear models for quintiles of the different indices. Furthermore, the agreement in ranking between the different indices was calculated by weighted kappa. All statistical analyses were conducted for boys and girls separately, and were adjusted for potential confounders.

**Results:**

Folate was positively associated with the *HFD*, the *HuSKY*, and fruit/vegetable intake for both boys and girls and with *IFI* for boys. Among girls, positive associations were seen between vitamin B_12_ and the *IFI* and between diastolic blood pressure and the *IFI* as well as fruit/vegetable intake. A negative association was found between homocysteine and the *HFD*, the *HuSKY*, and the *IFI* for both boys and girls and with fruit/vegetable intake for boys. Among boys, uric acid and *HbA1c* were negatively and prevalence of obesity positively associated with the *IFI*.

**Conclusions:**

Overall, the indices, even the simpler ones, seem to have a similar general capability in predicting biomarkers of dietary exposure. To predict risk of cardiovascular disease dietary indices may have to be more specific.

## Background

Adolescence is an important life stage for the establishment of health behaviour and could therefore also affect nutrition and health status later in life [1,2]. While longitudinal studies have indicated that biomarkers of cardiovascular disease predict biomarker values in adulthood [3-5], findings about the association of diet during adolescence and these biomarkers still remain inconsistent [3]. In the last decades, dietary indices have been used to study the relationship between food intake and disease [6-8]. The dietary index approach tries to account for the complex contribution of the human diet to health. Among adolescents, several indices to assess diet quality in adolescence exist [9-16], but literature concerning associations between such indices and biomarkers, which are used as indicators of the current health status, are rather sparse [17,18]. During the last years we developed several dietary indices, intended to reflect a healthy diet on basis of food based dietary guidelines for German children and adolescents [19]: the Healthy Food Diversity Index (*HFD*) [20], the Healthy Nutrition Score for Kids and Youth (*HuSKY*) [21], and the Indicator Food Index (*IFI*) [22]. Since the intake of fruits and vegetables is used as an indicator for a healthy diet in the national health monitoring in Germany, a simple index of fruit/vegetable intake was also developed. The aim of this study was to analyse the associations of these dietary indices with biomarkers of dietary exposure and cardiovascular status among adolescents in a nationally representative sample of German adolescents. Furthermore, the strength of the associations with biomarkers was compared for the mentioned indices.

## Methods

### Study design and study population

The nationally representative German Health Interview and Examination Survey for Children and Adolescents (*KiGGS*) was performed between 2003 and 2006 by the Robert Koch Institute. The aim of the *KiGGS* survey was to collect comprehensive data on the health status of children and adolescents aged 0 to 17 years. Participants were enrolled in two steps, in the first step, 167 sample points were randomly drawn stratified by federal state and community size. In the second step, participants were randomly selected from local population registries stratified by age. Children and adolescents with a migration background were also included. The final net sample included 17,641 participants, who lived in Germany [23]. The survey was approved by the German federal data protection office and by the ethics committee of Charité - University Medicine Berlin. Participants were informed in detail about the study objectives, interview and examination procedures as well as the handling of data records and analysis under pseudonymous conditions, and gave their written consent. Design and methods are described in detail elsewhere [24].

### Data collection and adaption of study variables

*KiGGS* includes several physical examinations, conducted by trained staff, among them blood pressure, body height and body weight measurements. Systolic and diastolic blood pressure (*BP*) were measured using an automated oscillometric device at an interval of two minutes. The arithmetic mean of two consecutive measurements was used for the analysis. Body height was measured according to a standardized protocol to the nearest 0.1 cm using a portable stadiometer. Body weight was measured in underwear to the nearest 0.1 kg with an electronic scale [24]. Since the body mass index (*BMI*) is dependent on growth related changes in body composition, a relative measure of *BMI* is more appropriate for adolescents. Therefore, *BMI* z-scores of the body mass index percentiles were calculated according to Schaffrath Rosario et al. [25]. For the analysis of biomarkers, blood samples were collected, separated into aliquots, frozen and stored at −40°C [26]. The fasting period was documented for every participant. To assess the usual intake of selected foods, a Food Frequency Questionnaire (*FFQ)* was used [27]. An energy intake index was calculated, summarising the multiplied amounts and mean energy contents of the *FFQ* items. Health-related behaviour like alcohol consumption, physical activity and smoking status of the participants were assessed with a self-administered questionnaire. Medication and supplement use of adolescents during the last seven days were determined with a standardised interview conducted by a physician [28]. Additionally, parents were asked about their income, occupational status and education. With this information a family socio-economic status index was calculated as described previously [29].

### Biochemical measures

In this study associations of dietary indices with biomarkers of long term nutrition were analysed. These biomarkers were selected from the biomarkers available for the study population. Since dietary indices are used to evaluate the accordance of an individual’s diet with nutritional recommendations, a positive association with biomarkers of dietary exposure (ferritin, *HbA1c*, folate, vitamin B_12_) was expected. Biomarkers of cardiovascular status (homocysteine, uric acid, C - reactive protein, total cholesterol, high-density lipoprotein cholesterol) were selected, which are predictive for levels in adulthood [2]. Homocysteine was determined with fluorescent particle immunoassay (Axsym; Abbott, Wiesbaden, Germany). Uric acid was determined by the uricase-PAP method (Hitachi 917; Roche, Mannheim, Germany). Total cholesterol was analysed using an enzymatic assay (cholesterol oxidase-PAP method) produced by Roche. High-density lipoprotein cholesterol (*HDL-C*) was determined directly with a homogenous enzymatic colorimetric assay (Roche). Serum C-reactive protein (*CRP*) was measured by Immunoturbidimetry (Hitachi 917). During the course of the survey the reagent produced by SCIL (Martinsried, Germany) was replaced by Roche. Due to parallel measurement, data derived with the SCIL reagent could be converted into a *CRP* value that corresponds with the new method. Serum vitamin B_12_, serum ferritin, and serum folate were measured by electrochemi luminescence immunoassay (Elecsys E2010; Roche). During the survey, the method for determination of folate was changed by the manufacturer. In the present analysis data obtained with both methods were analysed separately (first/second period), since conversion of the data was not feasible. *HbA1c* was analysed using high-performance liquid chromatography (Diastad; Biorad, Munich, Germany). Biochemical measures in the *KiGGS* study were described in detail elsewhere [26].

### Dietary assessment and construction of dietary indices

Usual consumption of several food groups during the “last few weeks” was assessed using a self-administered, semi quantitative *FFQ*[27]. The questionnaire was developed by the Robert Koch Institute and includes 45 food items. The frequency of consumption was assessed within ten categories, similar for all food items: never, once a month, two to three times a month, one to two times a week, three to four times a week, five to six times a week, one time per day, two to three times a day, four to five times a day, more than five times a day. In addition, participants had to estimate the usual portion size of the food item, which was given in five item specific categories. Several pictures were used to illustrate the portion sizes. The *FFQ* and a covering letter were sent by postal mail to the participants, several weeks prior the examination visit. The first page of the *FFQ* provides instructions about the completion of the questionnaire. Additionally, a telephone hotline was offered for any support in completing the questionnaire. At the examination visit the questionnaire was checked for completeness, and further support was offered. The *FFQ* was validated in comparison to the dietary history method DISHES and showed fair to moderate ranking validity for most food items (Spearman correlation coefficients from .35 to .69 with most values above .5), except for pasta/rice (.22) and white bread (.31) [30]. The validity of the *FFQ* is comparable to other *FFQs* for adolescents [30].

The food based dietary guidelines “Optimized Mixed Diet” (*OMD*) were developed to facilitate the adoption of a healthy diet to children and adolescents. The concept was described in detail elsewhere [19]. For this study, three dietary indices were selected, since these were developed especially for children and adolescents in Germany, taking into account the *OMD* recommendations. While the Healthy Nutrition Score for Kids and Youth (*HuSKY*) and the Indicator Food Index (*IFI*) were originally developed for *KiGGS*, the Healthy Food Diversity Index (*HFD*) was initially developed for adults and then adapted to adolescents. Additionally, for this study, a simple index of fruit and vegetable intake was calculated to compare with the more complex ones, since in the German national health monitoring these food groups are used as a main indicator of a healthy diet. For all dietary indices an increasing score is associated with a healthier diet.

### Healthy Nutrition Score for Kids and Youth (*HuSKY*)

The *HuSKY* was developed for the *KiGGS* study to compare eating habits of children and adolescents with the *OMD* guidelines [19]. To develop the index, 38 *FFQ* items were aggregated into eleven food groups corresponding to the guidelines [21]. Then, the ratio of food intake to food intake recommendation was calculated for each food group. On base of the sex and age-specific guidelines the ratio was allocated with points. For most food groups, intakes below the recommendation were proportionally allocated up to 100 points. If participants exceed the double recommended amount, points were proportionally subtracted from 100. The points of all food groups were summarized and afterwards, the *HuSKY* was standardized on a scale from 0 to 100. The *HuSKY* offers a valuable instrument to evaluate overall eating habits in a population, but is not intended to assess specific aspects of dietary behaviour in detail.

### Healthy Food Diversity Index (*HFD*)

The *HFD* was originally developed for the German Nutrition Survey (GeNuS) of 1998 among adults to assess the food diversity and the health value of an individual diet [20]. It considers three aspects: the number, distribution, and health value of all consumed foods. The index increases when the variation in food intake becomes healthier. Therefore the Berry-Index [31], which was applied in economic food diversity studies, was multiplied by a food-specific health factor based on the food consumption guidelines of the German Nutrition Society [32]. For our study, the *HFD* was adapted to adolescent’s diet. The intake of 41 *FFQ* items was used to calculate the index score. The food specific health factors were calculated according to the *OMD* guidelines [19]. Higher values of the *HFD* reflect a healthier diet. The consideration of both diversity and dietary recommendations seems to be the advantage of the *HFD*.

### Indicator Food Index (*IFI*)

A further, relatively simple index was developed previously in the research group [22]. Consumption of seven food groups of the *KiGGS FFQ* (fruits, vegetables, brown bread, soft drinks, fast food, chocolate, and salty snacks) was used as an indicator of a favourable or unfavourable diet. Therefore, frequency of each food group intake was categorized as healthy (2 points), neutral (1 point) and unfavourable (0 points). The points were defined using dietary guidelines and as a consensus of nutrition experts during a dietary indices expert meeting at a *KiGGS* symposium. By adding the points of all seven indicator food items an index with a scale from 0 to 14 was calculated. A score from 0 to 5 points was rated as an unfavourable, 6 to 10 points as a neutral and 11 to 14 points as a favourable diet. It should be emphasized, that this index covers only a few foods and therefore reflects only a selected proportion of the diet and not an overall dietary pattern. Furthermore, in comparison with *HFD* and *HuSKY* the estimation of health values for single food groups is relatively simple.

### Fruit/vegetable intake

As part of the continuous national health monitoring, the Robert Koch Institute regularly conducts telephone health interview surveys in representative samples of the German adult population (GEDA) [33]. Since the number of questions in a telephone interview is limited, only questions concerning the consumption of fruits, vegetables and fruit/vegetable beverages were included. These items can be used to build a simple indicator for a healthy diet but not to represent the general diet [34]. For the present study, a similar fruit/vegetable index was calculated to compare it with the more complex dietary indices. Standardised portions per day were calculated for six *FFQ* items (cooked, raw, frozen and tinned vegetables; fresh and tinned fruits). Subsequently, the portions were summarised. According to the nutritional recommendations of the German Nutrition Society up to one portion juice per day was added to fruit and vegetable consumption [32].

### Statistical analysis

For the present analyses we excluded participants without blood samples (N=292) and those, who did not completed the *FFQ* (N=263). Furthermore, pregnant participants were excluded from the analyses (N=2). Overall, out of 5,755 *KiGGS* participants our analyses included 5,198 participants. To avoid bias by medication use we excluded participants with diabetes and antidiabetic medication in the analyses for glycohaemoglobin (*HbA1c*; N=77). Furthermore, we excluded participants who used oral contraceptives in the analyses of serum lipids (N=432) and those who used oral contraceptives and antihypertensive medication in the analyses of blood pressure (N=468).

The sample of the *KiGGS* study was drawn by a clustered and stratified design, therefore all analyses were performed with complex-samples procedures of SPSS version 18.0 (SPSS Inc., Chicago, Illinois, USA). Since sex differences in dietary habits and pubertal status may be expected in this age group, we conducted separate analyses for boys and girls. To enhance representativeness for the German population structure, statistical analyses were weighted. For the comparison of the different dietary indices, scores were grouped into quintiles. Consequently the interpretation of the scales was similar for the indices, with higher quintiles indicating a healthier diet. The biomarkers values were generally not normally distributed. After a log transformation a normal distribution was also not achieved for all biomarkers but the results of the models did not change. Therefore the untransformed data are presented. Mean values of biomarkers with 95% confidence intervals were calculated according to quintiles of dietary indices, using complex-samples general linear model (CSGLM), and tested for trends. Additionally, regression coefficients for the association between biomarkers and indices were calculated by including the ranked index score as a continuous variable. All analyses were stratified for sex and adjusted for age (continuous), energy intake (continuous), *BMI* z-scores (continuous), alcohol consumption (yes, no), season of data collection (spring, summer, autumn, winter), physical activity (every day, 3–5 times/week, 1–2 times/week, 1–2 times/month, never), smoking status (yes, no), and family socio-economic index (low, medium, high status). The prevalence of obesity [35] was calculated for each quintile of the dietary indices. A trend test was conducted by logistic regression analysis, including the ranked index score as a continuous variable. Cronbach’s alpha [36], which is a function of the correlation and the number of items in a scale [37], was calculated to estimate the internal consistency of the dietary indices. The degree of agreement in ranking classification of the dietary indices was evaluated with calculation of the weighted kappa coefficient (κ_w_) using the formula [38]:

(1) $$\kappa_{w}=\frac{O_{w}-C_{w}}{1-C_{w}}$$

A cross table (5x5) of frequencies was calculated to derive the observed proportion of agreement (O_w_) and the expected proportion of agreement by chance (C_w_). The weighting factors were 1 for complete agreement, .75 for people differing one category, .5 for people differing two categories, .25 for people differing three categories, and 0 for complete disagreement. P-values less than .05 and non-overlapping 95% confidence intervals were considered statistical significant.

## Results

The study population is presented in Table 1 and includes boys (N=2,646) and girls (N=2,552) in nearly equal proportions. The mean age of all participants was 15.1, with a standard deviation of 1.7. Mean dietary index scores for girls were significantly higher than for boys, while energy intake for girls was lower than for boys. The prevalence of overweight and obesity was similar for both sexes. Alcohol consumption was significantly higher for boys than for girls, while smoking activity was similar for boys and girls. More boys (65%) than girls (42%) were physical active more than two times per week. Nearly 25% had a low or high socio-economic status and 50% had a medium socio-economic status. The survey was conducted during all seasons with some more examinations in autumn (30%) and winter (26%).

**Table 1:** Sample characteristics stratified for sex (mean values or percentages and 95% *CI*)*

	**All participants N=5,198**	**Boys N=2,646**	**Girls N=2,552**
Age	15.1	15.1 (15.0-15.1)	15.1 (15.0-15.1)
Energy intake	2,871	3,150 (3,089-3,211)	2,582 (2,529-2,634)
Index scores			
*HFD*	0.51	0.49 (0.48-.049)	0.54 (0.53-0.54)
*HuSKY*	53.1	51.8 (51.4-52.2)	54.5 (54.1-54.9)
*IFI*	9.03	8.58 (8.49-8.66)	9.50 (9.41-9.58)
Fruit/vegetable intake	3.30	3.02 (2.90-3.11)	3.59 (3.48-3.72)
Obesity status (%)^$^	17.2	17.2 (15.8-18.7)	17.2 (15.8-18.7)
Overweight	9.2	9.4 (8.4-10.6)	9.0 (7.9-10.1)
Obese	8.0	7.8 (6.8-8.9)	8.2 (7.2-9.3)
Alcohol consumption (%)	22.9	29.0 (27.3-30.7)	16.5 (15.1-18.0)
Smoking (%)	22.5	22.3 (20.7-23.9)	22.7 (20.8-23.9)
Physical activity (%)			
Every day	21.6	27.1 (25.5-28.9)	15.8 (14.4-17.2)
3-5times/week	32.2	37.9 (36.1-39.8)	26.2 (24.5-27.9)
1-2times/week	30.6	25.2 (23.6-26.9)	36.2 (34.4-38.1)
1-2times/day	5.5	3.8 (3.1-6.4)	7.3 (6.4-8.4)
Never	10.1	5.9 (5.0-6.9)	14.5 (19.1-22.2)
*SES* (%)^$$^			
Low	25.8	25.9 (24.3-27.6)	25.7 (24.0-27.4)
Medium	48.1	47.6 (45.7-49.5)	48.7 (46.8-50.6)
High	26.0	26.5 (24.8-28.2)	25.6 (24.0-27.4)
Season (%)			
Spring	21.9	22.4 (20.8-24.0)	21.4 (19.9-23.1)
Summer	21.8	21.8 (20.3-23.4)	21.8 (20.2-23.4)
Autumn	30.3	30.2 (28.5-32.0)	30.3 (28.5-32.1)
Winter	26.0	25.6 (24.0-27.3)	26.5 (24.8-28.2)

Abbreviation: *CI* (Confidence interval), *HFD* (Healthy Food Diversity Index), *HuSKY* (Healthy Nutrition Score for Kids and Youth), *IFI* (Indicator Food Index), *SES* (Socio-economic status).

*Non-overlapping 95% confidence intervals were considered statistical significant.

^$^calculated according to Kromeyer-Hauschild et al. [35].

^$$^calculated according to Winkler and Stolzenberg [29].

### Dietary indices and biomarkers of dietary exposure

The association of dietary indices with biomarkers of dietary exposure for girls are presented in Table 2. Mean values of folate increased for increasing quintiles of the *HFD* (p=.010/<.001) and the fruit/vegetable intake (p=.017/.044) in both study periods. For the *HuSKY*, mean values of folate increased only in the second study period. The mean value of vitamin B_12_ increased across increasing quintiles of the *IFI* (p=.003). The association of dietary indices with biomarkers of dietary exposure for boys are presented in Table 3. Borderline significant decreasing *HbA1c* values were observed across increasing quintiles of the *IFI* (p=.049). Mean values of folate increased across increasing quintiles for all indices, within the first study period. The strongest association showed the *IFI* (p=.006). The results for the second study period showed only in tendency a linear association between folate and the indices.

**Table 2:** **Biomarkers**^**# **^of dietary exposure by quintiles of dietary indices for girls (mean and 95% *CI*)*

	**Index**	**1. Quintile**	**3. Quintile**	**5. Quintile**	**ß**	**p**
Ferritin μg/l	*HFD*	35.2	32.6	33.5	−0.354	.328
N=2,523		32.8-37.5	30.1-35.1	31.1-36.0		
	*HuSKY*	35.1	34.0	33.7	−0.183	.589
		32.6-37.5	31.4-36.6	31.3-36.1		
	*IFI*	33.9	34.8	34.1	−0.127	.782
		31.3-36.5	31.7-37.9	31.4-36.7		
	Fruit/vegetable intake	33.0	34.6	33.6	0.240	.529
		30.4-35.7	31.5-37.8	31.3-35.9		
*HbA1c* %	*HFD*	4.80	4.84	4.81	−0.002	.891
N=2,494		4.74-4.86	4.79-4.89	4.76-4.85		
	*HuSKY*	4.84	4.80	4.80	−0.009	.212
		4.78-4.90	4.75-4.86	4.74-4.85		
	*IFI*	4.83	4.80	4.83	0.005	.528
		4.77-4.88	4.74-4.85	4.78-4.89		
	Fruit/vegetable intake	4.83	4.83	4.80	−0.011	.136
		4.77-4.88	4.77-4.89	4.75-4.86		
Folate (First period) ng/ml	*HFD*	450.7	482.6	490.5	9.859	**.010**
N=1,502		423.6-477.8	457.9-507.3	463.5-517.5		
	*HuSKY*	468.8	466.8	471.8	−0.929	.786
		442.1-495.6	434.4-499.2	448.0-495.6		
	*IFI*	471.6	458.0	490.0	4.700	.142
		446.0-497.2	434.9-481.1	460.9-519.1		
	Fruit/vegetable intake	435.4	482.3	489.4	7.814	**.017**
		410.6-460.3	457.0-507.6	464.8-514.0		
Folate (Second period) ng/ml	*HFD*	608.5	668.7	675.9	15.784	**<.001**
N=947		570.2-646.8	630.9-706.5	649.1-702.8		
	*HuSKY*	622.3	665.4	675.6	13.293	**<.001**
		587.0-657.7	624.8-706.1	671.4-709.5		
	*IFI*	629.9	670.6	648.8	8.685	.094
		593.5-666.3	634.8-706.4	620.5-677.1		
	Fruit/vegetable intake	624.4	643.1	679.0	9.936	**.044**
		582.9-665.8	607.3-679.0	643.4-714.6		
Vitamin B_12_ ng/1	*HFD*	445.0	485.7	457.0	0.962	.455
N=2,518						
		421.4-468.5	463.5-507.8	436.4-477.6		
	*HuSKY*	474.8	458.3	471.5	−0.751	.857
		443.6-505.7	432.2-484.4	445.8-497.3		
	*IFI*	444.9	488.4	486.1	10.870	**.003**
		419.9-469.9	457.6-519.1	460.7-511.5		
	Fruit/vegetable intake	479.9	464.7	458.8	−3.606	.060
		453.9-505.9	437.6-491.9	436.0-481.6		

Abbreviation: *CI* (Confidence interval), *HbA1c* (Glycohaemoglobin), *HFD* (Healthy Food Diversity Index), *HuSKY* (Healthy Nutrition Score for Kids and Youth), *IFI* (Indicator Food Index).

*P values less than 0.05 (bold) were considered statistically significant.

^#^adjusted for age, energy intake, *BMI*, alcohol consumption, season, physical activity, smoking status, and family socio-economic index.

**Table 3:** **Biomarkers**^**# **^of dietary exposure by quintiles of dietary indices for boys (mean and 95% *CI*)*

	**Index**	**1. Quintile**	**3. Quintile**	**5. Quintile**	**ß**	**p**
Ferritin μg/l	*HFD*	53.3	52.2	58.7	0.808	.109
N=2,630		49.9-56.7	48.6-55.7	52.7-64.8		
	*HuSKY*	53.2	53.4	59.4	0.806	.130
		49.3-57.2	49.7-57.2	52.8-66.0		
	*IFI*	53.2	52.6	55.8	0.646	.136
		49.2-57.2	48.6-56.6	50.9-60.7		
	Fruit/vegetable intake	54.3	53.3	53.3	−0.227	.808
		50.6-58.0	49.6-57.0	47.2-59.5		
*HbA1c* %	*HFD*	4.93	4.89	4.89	−0.010	.106
N=2,592		4.89-4.97	4.84-4.93	4.83-4.94		
	*HuSKY*	4.91	4.92	4.88	−0.002	.574
		4.86-4.96	4.87-4.96	4.82-4.94		
	*IFI*	4.91	4.92	4.86	−0.012	**.049**
		4.86-4.95	4.87-4.98	4.78-4.93		
	Fruit/vegetable intake	4.91	4.91	4.89	0.003	.677
		4.87-4.96	4.86-4.96	4.82-4.95		
Folate (First period) ng/ml	*HFD*	462.3	481.2	507.1	10.260	**.009**
		436.3-488.2	456.6-505.8	475.2-539.0		
N=1,604						
	*HuSKY*	460.7	457.0	493.2	6.801	**.030**
		434.6-486.8	429.0-484.9	463.1-523.3		
	*IFI*	451.1	465.0	501.0	27.671	**.006**
		429.4-472.7	438.3-491.6	464.3-537.8		
	Fruit/vegetable intake	446.3	464.0	496.0	10.057	**.005**
		422.5-470.0	437.0-491.0	466.9-525.1		
Folate (Second period) ng/ml	*HFD*	625.6	677.2	630.4	0.860	.949
N=953		592.7-658.5	646.9-707.5	586.5-674.3		
	*HuSKY*	638.7	643.5	656.8	3.095	.502
		605.4-672.0	615.1-671.9	619.7-693.9		
	*IFI*	635.6	658.9	639.6	3.692	.763
		603.4-667.7	625.4-692.3	598.1-681.1		
	Fruit/vegetable intake	638.4	672.2	663.4	6.901	.185
		608.9-667.8	641.8-702.6	625.4-701.4		
Vitamin B_12_ ng/l	*HFD*	471.6	476.9	477.6	1.464	.766
N=2,609		451.3-491.8	454.2-499.7	450.1-505.0		
	*HuSKY*	487.0	488.7	484.3	−3.660	.742
		459.0-514.9	462.8-514.7	451.4-517.2		
	*IFI*	474.2	499.1	477.7	6.227	.259
		451.4-497.0	478.0-520.2	446.4-509.0		
	Fruit/vegetable intake	486.3	473.8	474.2	−1.992	.569
		463.0-509.5	453.3-494.4	446.4-502.0		

Abbreviation: *CI* (Confidence interval), *HbA1c* (Glycohaemoglobin), *HFD* (Healthy Food Diversity Index), *HuSKY* (Healthy Nutrition Score for Kids and Youth), *IFI* (Indicator Food Index).

*P values less than 0.05 (bold) were considered statistically significant.

#adjusted for age, energy intake, *BMI*, alcohol consumption, season, physical activity, smoking status, and family socio-economic index.

### Dietary indices and biomarkers of cardiovascular status

The association of dietary indices with biomarkers of cardiovascular status for girls are presented in Table 4. Mean values of homocysteine decreased across increasing quintiles of the *HFD*, the *HuSKY*, and the *IFI*. The strongest association was observed for the *HuSKY* (p=.007) and the *IFI* (p=.027). Mean values of *CRP* decreased significantly across increasing quintiles for the *IFI* (p=.007). Mean values of the diastolic *BP* increased significantly across increasing quintiles of the *IFI* (p=.018) and fruit/vegetable intake (p=.046). The association of dietary indices with biomarkers of cardiovascular status for boys are presented in Table 5. Mean values of homocysteine decreased across increasing quintiles of the *HFD*, the *HuSKY*, the *IFI*, and fruit/vegetable intake. The strongest associations were found for the *IFI* (p=.001). Additionally, mean values of uric acid decreased for increasing quintiles of the *HFD* (p=.001) and the *IFI* (p<.001). Mean values of *CRP* decreased significantly across increasing quintiles for the *HuSKY* (p=.034).

**Table 4:** **Biomarkers**^**# **^of cardiovascular status by quintiles of dietary indices for girls (mean and 95% *CI*)*

	**Index**	**1. Quintile**	**3. Quintile**	**5. Quintile**	**ß**	**p**
Homocysteine μmol/l	*HFD*	8.09	7.88	7.69	−0.074	**.038**
N=2,522		7.80-8.39	7.60-8.16	7.49-7.89		
	*HuSKY*	8.37	7.88	7.83	−0.108	**.007**
		8.03-8.71	7.62-8.14	7.58-8.09		
	*IFI*	8.11	8.01	7.71	−0.098	**.027**
		7.83-8.39	7.74-8.28	7.42-7.99		
	Fruit/vegetable intake	8.13	7.84	7.79	−0.058	.165
		7.82-8.45	7.59-8.08	7.50-8.08		
Uric acid mg/dl	*HFD*	4.28	4.26	4.34	0.015	.336
N=2,537		4.18-4.38	4.15-4.37	4.25-4.43		
	*HuSKY*	4.39	4.26	4.30	−0.011	.331
		4.29-4.50	4.15-4.37	4.21-4.40		
	*IFI*	4.25	4.31	4.37	0.027	.099
		4.14-4.35	4.20-4.43	4.25-4.48		
	Fruit/vegetable intake	4.27	4.33	4.38	0.024	.182
		4.16-4.38	4.22-4.43	4.27-4.50		
*CRP* μg/dl	*HFD*	196.1	151.8	162.5	−6.073	.175
N=2,438		148.7-243.5	115.7-187.9	123.7-201.4		
	*HuSKY*	193.9	166.5	154.9	−8.215	.051
		150.4-237.4	112.5-220.5	115.9-194.0		
	*IFI*	205.8	164.6	155.5	−14.005	**.007**
		148.1-263.6	121.3-207.9	112.7-198.4		
	Fruit/vegetable intake	178.4	158.2	154.8	−2.283	.586
		137.1-219.7	119.4-197.0	116.5-193.2		
Total cholesterol μg/dl	*HFD*	158.9	160.6	161.6	0.634	.190
N=2,104		155.1-162.7	156.9-164.2	157.9-165.3		
	*HuSKY*	160.6	162.0	160.5	−0.033	.930
		156.5-164.7	158.2-165.8	156.6-164.3		
	*IFI*	161.6	162.3	161.4	0.033	.790
		157.8-165.4	158.4-166.2	157.4-165.4		
	Fruit/vegetable intake	159.9	160.1	158.8	0.099	.869
		155.8-163.9	155.5-164.6	154.8-162.8		
*HDL-C* μg/dl	*HFD*	58.2	57.5	57.3	−0.359	.108
N=2,104		56.5-60.0	56.0-59.0	55.8-58.8		
	*HuSKY*	58.4	58.1	56.7	−0.278	.205
		56.8-60.0	56.5-59.7	55.3-58.1		
	*IFI*	58.2	57.3	57.5	−0.184	.418
		56.3-60.1	55.7-58.8	56.2-58.9		
	Fruit/vegetable intake	58.3	57.0	56.9	−0.202	.291
		56.6-60.1	55.4-58.6	55.5-58.2		
Systolic *BP* mmHg	*HFD*	111.8	112.0	112.9	0.223	.205
N=2,100		110.2-113.3	110.7-113.3	111.7-114.1		
	*HuSKY*	111.8	112.7	112.6	0.057	.287
		110.4-113.1	111.5-113.9	111.4-113.9		
	*IFI*	112.2	111.2	113.4	0.394	.052
		110.8-113.6	109.9-112.4	112.0-114.8		
	Fruit/vegetable intake	111.6	113.3	112.1	0.099	.485
		110.2-113.0	111.8-114.7	110.8-113.4		
Diastolic *BP* mmHg	*HFD*	66.5	67.6	67.7	0.128	.235
N=2,100						
		65.7-67.4	66.7-68.4	66.8-68.6		
	*HuSKY*	67.5	67.6	67.8	0.096	.564
		66.6-68.3	66.7-68.4	66.9-68.8		
	*IFI*	67.2	66.5	68.3	0.326	**.018**
		66.3-68.0	65.5-67.5	67.3-69.3		
	Fruit/vegetable intake	66.7	67.7	67.8	0.255	**.046**
		65.8-67.5	66.7-68.8	66.8-68.8		

Abbreviation: *BP* (Blood pressure), *CI* (Confidence interval), *CRP* (C - reactive protein), *HDL-C* (high-density lipoprotein cholesterol), *HFD* (Healthy Food Diversity Index), *HuSKY* (Healthy Nutrition Score for Kids and Youth), *IFI* (Indicator Food Index).

*P values less than 0.05 (bold) were considered statistically significant.

^#^adjusted for age, energy intake, *BMI*, alcohol consumption, season, physical activity, smoking status, and family socio-economic index.

**Table 5:** **Biomarkers**^**# **^of cardiovascular status by quintiles of dietary indices for boys (mean and 95% *CI*)*

	**Index**	**1. Quintile**	**3. Quintile**	**5. Quintile**	**ß**	**P**
Homocysteine μmol/l	*HFD*	9.48	9.20	8.77	−0.164	**.001**
N=2,625		9.03-9.93	8.78-9.63	8.32-9.21		
	*HuSKY*	9.69	9.09	8.89	−0.147	**.018**
		9.19-10.19	8.65-9.53	8.45-9.32		
	*IFI*	9.84	9.40	8.79	−0.250	**.001**
		9.33-10.35	8.92-9.89	8.27-9.31		
	Fruit/vegetable intake	9.83	9.05	9.20	−0.189	**.037**
		9.39-10.28	8.67-9.42	8.61-9.79		
Uric acid mg/dl	*HFD*	5.72	5.46	5.45	−0.068	**.001**
N=2,635		5.60-5.84	5.35-5.57	5.31-5.59		
	*HuSKY*	5.58	5.48	5.52	−0.020	.273
		5.47-5.70	5.36-5.60	5.38-5.66		
	*IFI*	5.64	5.57	5.31	−0.064	**<.001**
		5.53-5.75	5.44-5.69	5.16-5.46		
	Fruit/vegetable intake	5.56	5.58	5.59	0.001	.916
		5.44-5.69	5.47-5.69	5.45-5.73		
*CRP* μg/dl	*HFD*	130.0	109.0	120.3	−3.846	.430
N=2,554		95.4-164.6	82.5-135.5	83.2-157.4		
	*HuSKY*	124.5	143.6	108.8	−8.205	**.034**
		99.1-149.9	107.5-179.6	79.5-138.1		
	*IFI*	132.3	112.5	123.1	−5.034	.528
		104.9-159.7	84.9-140.1	86.5-159.6		
	Fruit/vegetable intake	133.1	126.2	127.8	−2.528	.654
		103.0-163.1	87.3-165.1	92.7-162.9		
Total cholesterol μg/dl	*HFD*	153.5	154.7	154.4	−0.044	.842
N=2,634		150.6-156.4	151.0-158.3	150.5-158.3		
	*HuSKY*	153.9	156.8	154.4	0.372	.538
		150.7-157.0	153.2-160.4	150.8-158.4		
	*IFI*	153.9	156.5	155.0	0.414	.559
		150.8-157.0	152.8-160.1	150.6-159.5		
	Fruit/vegetable intake	154.8	157.4	153.0	−0.225	.533
		151.4-158.2	154.3-160.5	148.7-157.2		
*HDL-C* μg/dl	*HFD*	52.0	53.4	52.9	0.144	.557
N=2,634		50.8-53.3	52.1-54.8	51.3-54.4		
	*HuSKY*	54.2	52.9	52.2	−0.374	.051
		52.9-55.5	51.4-54.3	50.7-53.8		
	*IFI*	53.1	52.7	51.7	−0.148	.218
		52.0-54.3	51.3-54.1	50.1-53.4		
	Fruit/vegetable intake	53.3	53.3	51.7	−0.271	.143
		52.0-54.6	52.1-54.5	50.1-53.3		
Systolic *BP* mmHg	*HFD*	117.4	117.5	118.3	0.100	.552
N=2,624		116.3-118.5	116.1-119.0	116.9-119.7		
	*HuSKY*	118.3	117.9	117.5	−0.224	.221
		117.1-119.4	116.7-119.1	116.2-118.9		
	*IFI*	117.1	118.5	117.9	0.309	.265
		116.1-118.1	117.3-119.8	116.2-119.6		
	Fruit/vegetable intake	118.1	118.0	118.1	0.011	.865
		116.8-119.3	117.0-119.1	116.5-119.6		
Diastolic *BP* mmHg	*HFD*	69.3	68.7	69.5	−0.018	.991
N=2,624		68.5-70.1	67.7-69.7	68.3-70.6		
	*HuSKY*	69.4	69.1	69.1	−0.087	.599
		68.6-70.3	68.2-70.0	68.0-70.2		
	*IFI*	69.0	69.4	70.2	0.247	.121
		68.3-69.7	68.5-70.3	68.6-71.8		
	Fruit/vegetable intake	69.3	69.7	69.4	−0.015	.981
		68.5-70.1	68.9-70.6	68.1-70.6		

Abbreviation: *BP* (Blood pressure), *CI* (Confidence interval), *CRP* (C - reactive protein), *HDL-C* (high-density lipoprotein cholesterol), *HFD* (Healthy Food Diversity Index), *HuSKY* (Healthy Nutrition Score for Kids and Youth), *IFI* (Indicator Food Index).

*P values less than 0.05 (bold) were considered statistically significant.

^#^adjusted for age, energy intake, *BMI*, alcohol consumption, season, physical activity, smoking status, and family socio-economic index.

Table 6 shows the prevalence of obesity according to quintiles of dietary indices stratified for sex. There is a tendency to higher percentages of obesity for higher quintiles of dietary indices. Only for the *IFI* among boys, the trend was significant.

**Table 6:** **Obese adolescents**^**$ **^(percentages and 95% *CI*) according to quintiles of dietary indices*

	**Index**	**1. Quintile**	**3. Quintile**	**5. Quintile**	**ß**	**p**
Girls	*HFD*	20.5	17.1	16.9	−0.061	.162
N=2,544		16.7-24.6	14.0-20.6	14.1-20.0		
	*HuSKY*	16.2	16.2	18.7	0.063	.159
		12.9-20.0	13.1-19.8	15.8-22.0		
	*IFI*	12.7	20.3	18.4	0.078	.092
		9.8-16.1	16.5-24.5	14.9-22.3		
	Fruit/vegetable intake	18.8	18.7	19.2	0.044	.382
		15.2-22.9	15.3-22.4	16.1-22.6		
Boys	*HFD*	14.0	17.6	17.6	0.041	.364
N=2,634		11.4-16.9	14.5-21.1	14.1-21.6		
	*HuSKY*	15.9	18.0	19.2	0.051	.267
		13.0-19.1	14.8-21.5	15.4-23.4		
	*IFI*	13.6	19.4	21.0	0.158	**.001**
		11.4-16.2	15.9-23.3	16.4-26.3		
	Fruit/vegetable intake	19.0	15.7	19.6	0.009	.847
		15.9-22.3	12.8-19.0	15.9-23.7		

Abbreviation: *CI* (Confidence interval), *HFD* (Healthy Food Diversity Index), *HuSKY* (Healthy Nutrition Score for Kids and Youth), *IFI* (Indicator Food Index).

*P values less than 0.05 (bold) were considered statistically significant.

^$^calculated according to Kromeyer-Hauschild et al. [35].

The values of Cronbach’s alpha are similar for the *HuSKY* (.81) and *HFD* (.80), while values for fruit/vegetable intake (.67) and *IFI* (.62) are somewhat lower. To compare ranking agreement of the dietary indices weighted kappa coefficients were calculated. Most indices showed coefficients between .30 and .35 in ranking of participants. The *HuSKY* index and fruit/vegetable intake showed the highest agreement (κ_w_=.42). Spearman’s correlation coefficients showed similar results (data not presented).

## Discussion

Among German adolescents, the higher quintiles of the *HFD*, *HuSKY*, *IFI* and fruit/vegetable intake were associated with a more favourable biomarker profile, including higher ferritin, higher folate, higher vitamin B_12_; lower *HbA1c*, lower homocysteine, lower uric acid and lower *CRP* mean values. Most significant associations between dietary indices and biomarkers were observed for folate and homocysteine. Overall, the biomarkers of cardiovascular status showed less significant associations with the dietary indices than biomarkers of dietary exposure.

### Dietary indices and biomarkers of dietary exposure

Serum levels of folate, *HbA1c*, Ferritin, and vitamin B_12_ are indicators of the current status for specific nutrients. Furthermore, folate, ferritin and vitamin B_12_ were associated with the risk of developing chronic diseases [39,40]. Considering biomarkers of dietary exposure, most significant associations with dietary indices were found for folate. This may be due to the fact, that fruits and leafy vegetables, which are the main sources of folate [41], have a clear positive impact in all examined indices. The main sources of vitamin B_12_ are animal source foods like meat and dairy products [41,42]. Since intake of these products is generally high in Germany, a diet rich in those will reduce the index values of *HFD* and *HuSKY* and may therefore weaken the association with vitamin B_12_. The two simpler indices do not contain these animal source food groups. The main source of heme iron, a metabolic precursor of ferritin, is red meat [43]. Overall, the dietary indices may show no significant association with ferritin, since high meat consumption either reduces the score or meat consumption is not included. Furthermore, both *HFD* and *HuSKY* do not distinct between different heme contents of meat sources (beef vs. poultry). *HbA1c* is a biomarker of long-term glycaemic control and it is used as a diabetes screening indicator [44]. Diets, which are low in whole grains or dietary fibre may increase *HbA1c* values and are associated with a higher risk of type 2 diabetes [45]. Only the *IFI* was significantly associated to *HbA1c*, among boys. This is not surprising, since the analysed indices, except the *IFI*, do not account for fibre content. Overall, the dietary indices seem to be useful to predict serum concentration of folate. Biomarkers which are associated with intake of meat or dietary fibre are not well reflected by these indices of an overall healthy diet. Other studies among adults observed similar associations [46-48].

### Dietary indices and biomarkers of cardiovascular status

In previous studies, blood concentrations of homocysteine [5], uric acid [49], *CRP*[50], blood pressure and blood lipids [51] were associated with cardiovascular disease risk. Serum total cholesterol shows a positive relationship with cardiovascular disease risk whereas *HDL-C* is inversely related. Overall, independent of other factors like physical activity, the risk of cardiovascular disease may increase by high consumption of saturated fats, salt and refined carbohydrates, as well as low consumption of fruits and vegetables [5]. As described above, the analysed indices are based on a relatively simple *FFQ* (45 food items) and rank the diet of participants according to a consensus of an overall healthy diet. In the indices, for instance, saturated fats, salt and fibre intake are not well reflected and most associations between dietary indices and biomarkers of cardiovascular status are not significant. However, in most cases dietary indices and biomarkers of cardiovascular risk are in tendency inversely associated. The associations between dietary indices and homocysteine were most often significant. This result is not surprising, since all analysed indices are positively associated to serum concentrations of folate and folate is required for metabolism of homocysteine to methionine [52]. High intake of carbohydrates may increase blood levels of *CRP*[53]. All analysed indices showed in tendency a negative association with *CRP*. Only among girls, the association with *IFI* was significant. This may be due to the fact that the *IFI* score increases when consumption of brown bread is high but decreases when consumption of other grain sources, like fast food and salty snacks, is high. A modified version of the *IFI* may better predict carbohydrate intake and *CRP* values. The association with *HbA1c*, the long term marker of carbohydrate intake, was only significant for *IFI*, among boys. The main sources of cholesterol are dairy fat and meat [5]. The fact that fat content of dairy products was not accounted for in the index scores, could be a reason why no significant association was observed for dietary indices and blood lipids (total cholesterol and *HDL-C*). Low fat milk and milk products lower the risk of hypertension [54]. Among girls, diastolic blood pressure increased significantly with increasing index scores for *IFI* and fruit/vegetable intake. The reason may be that milk and milk products are not included in the calculation of *IFI* and fruit/vegetable intake. Overall, the biomarkers of cardiovascular status showed less significant associations with the indices than biomarkers of dietary exposure. This may be because the intake of the relevant food groups for cardiovascular disease risk is not well represented in the analysed indices. As mentioned above, studies concerning the association of dietary indices and biomarkers of cardiovascular status are rather sparse. Among Cypriot children [17] increasing *CRP* levels were significantly associated with a dietary inflammation index, since this index included fried foods, sweets, junk and fatty foods. In contrary to our findings the E-KINDEX score [18], an index that has been developed to identify children, whose dietary habits can predict obesity, showed a significant negative association with blood pressure. Among adults, several studies observed an inverse association between dietary indices and biomarkers of cardiovascular status [55-57]. Maybe, adolescents are too young to observe already clear associations between dietary indices and biomarkers of cardiovascular disease status.

### Influence of other factors

The onset of puberty may influence dietary habits, food intake reporting and biomarker values. Girls as well as boys may reduce their food intake or misreport consumption because of weight concerns [58]. Additionally, levels of biomarkers of cardiovascular disease are associated with the onset of puberty [3]. Considering that in general puberty starts at different ages in girls and boys [59] this may have an impact on sex differences. For example, among girls at pubertal age *HDL-C* levels are higher than among boys of the same age [60]. These differences may result in effect modification of the associations and therefore the analyses were stratified for sex.

Obesity is a risk factor for cardiovascular disease and has influence on serum levels of biomarkers e.g. *CRP*[17], cholesterol and it is associated with hypertension [61]. For all dietary indices, there was a tendency for higher proportions of obese adolescents with increasing quintiles of indices. This association seems unexpected, but this is observed for many dietary indices [62]. A part of the explanation is that people, who consume higher amounts of food, tend to meet the recommendation for adequate intake more often than people, who eat less food. Therefore persons, who eat more, tend to have higher scores. Because of the mentioned associations, analyses between dietary indices and biomarkers were adjusted for *BMI*.

### Agreement of dietary indices

The analysed dietary indices differ in the underlying assumptions of what characterises a healthy diet, which is reflected in the poor range of the weighted kappa coefficients between the indices. For example, the *HuSKY* index focusses on the accordance with quantities of recommended food intake of the *OMD* guidelines, while the *HFD* additionally takes the diversity of diet into account. The fair agreement between the *HuSKY* index and fruit/vegetable intake may be due to the importance of fruit and vegetable intake for the *HuSKY* score. For most food items, points were proportionally subtracted from 100 when intake exceeds the double recommended amount, but the intake of fruits and vegetables is allocated with 100 points either if a participant reaches or exceeds the recommendation. The internal consistency of the dietary indices is comparable to the reported reliability of other dietary scales among adolescents [18] and adults [63,64]. Somewhat lower values of Cronbach’s alpha for the IFI and Fruit/vegetable intake may be due to the small number of food items. However, these simple tools may be useful in large population studies.

### Study limitations and methodological considerations

Some limitations of the present study must be acknowledged. The cross-sectional study design may be a limitation for the estimation of the true association between indices and biomarkers of cardiovascular status, since the biomarkers are affected by long-term diet. This may blur the association and biomarkers of cardiovascular status may show less significant associations with the dietary indices. Furthermore, the diet was assessed by a *FFQ* with its well-known limitations [65]. Dietary indices are used to evaluate a healthy diet by calculating a one-dimensional index score. Since the overall diet of an individual is characterised by many aspects, like meal structure, foods consumed and frequency of consumption, one single value may reflect many different dietary patterns [66]. Biomarker values are an objective tool to evaluate the dietary status and disease risk. However, especially the association between biomarkers of cardiovascular status and cardiovascular disease need to be critically evaluated [49]. For example, the elevation of plasma homocysteine may be rather a consequence than a cause of atherosclerosis.

We adjusted our models for possible confounders of the association between dietary variables and biomarkers. However, some biomarkers are influenced by other factors, which could not be completely considered. For example, both physical activity and energy intake may confound the observed association between a healthy diet and blood pressure. In this survey physical activity and energy intake were assessed from self-reports and allow only a rough estimate. Similar, the association between dietary indices and biomarker values may be influenced by supplement use. Since only data on supplement use of the last seven days are available, corrections for the associations with biomarkers of long term nutrition could be insufficient.

## Conclusions

Not many studies investigated the association of dietary indices and biomarkers of dietary exposure and cardiovascular status among adolescents. This study is based on a large representative sample of German adolescents. The associations with dietary indices were most pronounced for folate and homocysteine. Overall, the indices, even the simpler ones, may have a similar general capability in predicting biomarkers of dietary exposure. The biomarkers of cardiovascular status showed less significant associations with the indices. To predict risk of cardiovascular disease dietary indices have to be more specific, for instance with regard to specific intakes of meat and dairy products. Other foods, which are not relevant for a specific outcome, may be excluded.

## Abbreviations

BMI: Body mass index; BP: Blood pressure; CRP: C-reactive protein; DGE: German Nutrition Society; FFQ: Food Frequency Questionnaire; HbA1c: Glycohaemoglobin; HDL-C: High-density lipoprotein cholesterol; HFD: Healthy Food Diversity Index; HuSKY: Healthy Nutrition Score for Kids and Youth; IFI: Indicator Food Index; KiGGS: German Health Interview and Examination Survey for Children and Adolescents; OMD: Optimised mixed diet.

## Competing interests

The authors declare that they have no competing interests.

## Authors' contributions

JT conducted the present analysis and prepared the manuscript. GBMM and AR assisted with statistical analysis. GBMM, AR, ST, LD and JR contributed to the interpretation of the results and the writing of the manuscript. GBMM was involved in the design of the KiGGS study and is the major responsible developer of the KiGGS FFQ. All the authors were involved in the critical revision of the manuscript.

## References

[B1] Lake AA Mathers JC Rugg-Gunn AJ Adamson AJ Longitudinal change in food habits between adolescence (11–12 years) and adulthood (32–33 years): the ASH30 Study J Publ Health 2006 28 10 16 10.1093/pubmed/fdi082 16473923

[B2] Kelder SH Perry CL Klepp KI Lytle LL Longitudinal tracking of adolescent smoking, physical activity, and food choice behaviors Am J Public Health 1994 84 1121 1126 10.2105/AJPH.84.7.1121 8017536 PMC1614729

[B3] Day RS Fulton JE Dai S Mihalopoulos NL Barradas DT Nutrient intake, physical activity, and CVD risk factors in children: project heartbeat! Am J Prev Med 2009 37 S25 S33 10.1016/j.amepre.2009.04.006 19524152 PMC2729283

[B4] Berenson GS Srnivasan SR Cardiovascular risk factors in youth with implications for aging: The Bogalusa Heart Study Neurobiol Aging 2005 26 303 307 10.1016/j.neurobiolaging.2004.05.009 15639307

[B5] World Health Organization, Food Agriculture Organization of the United Nations Diet, nutrition, and the prevention of chronic diseases: report of a joint WHO/FAO expert consultation 2003 World Health Organization

[B6] Kant AK Indexes of overall diet quality: a review J Am Diet Assoc 1996 96 785 791 10.1016/S0002-8223(96)00217-9 8683010

[B7] Hu FB Dietary pattern analysis: a new direction in nutritional epidemiology Curr Opin Lipidol 2002 13 3 9 10.1097/00041433-200202000-00002 11790957

[B8] Arvaniti F Panagiotakos DB Healthy indexes in public health practice and research: a review Crit Rev Food Sci Nutr 2008 48 317 327 10.1080/10408390701326268 18409114

[B9] Golley RK Hendrie GA McNaughton SA Scores on the dietary guideline index for children and adolescents are associated with nutrient intake and socio-economic position but not adiposity J Nutr 2011 141 1340 1347 10.3945/jn.110.136879 21613454

[B10] Acar Tek N Yildiran H Akbulut G Bilici S Koksal E Gezmen Karadag M Sanlıer N Evaluation of dietary quality of adolescents using Healthy Eating Index Nutr Res Pract 2011 5 322 328 10.4162/nrp.2011.5.4.322 21994527 PMC3180683

[B11] Feskanich D Rockett HRH Colditz GA Modifying the healthy eating index to assess diet quality in children and adolescents J Am Diet Assoc 2004 104 1375 1383 10.1016/j.jada.2004.06.020 15354153

[B12] Mirmiran P Azadbakht L Azizi F Dietary quality-adherence to the dietary guidelines in Tehranian adolescents: Tehran lipid and glucose study Int J Vitam Nutr Res 2005 75 195 200 10.1024/0300-9831.75.3.195 16028635

[B13] Hurley KM Oberlander SE Merry BC Wrobleski MM Klassen AC Black MM The healthy eating index and youth healthy eating index are unique, nonredundant measures of diet quality among low-income, African American adolescents J Nutr 2009 139 359 364 19074210 10.3945/jn.108.097113 PMC2646206

[B14] Huybrechts I Vereecken C De Bacquer D Vandevijvere S Van Oyen H Maes L Vanhauwaert E Temme L De Backer G De Henauw S Reproducibility and validity of a diet quality index for children assessed using a FFQ Br J Nutr 2010 104 135 144 10.1017/S0007114510000231 20214836

[B15] Angelopoulos P Kourlaba G Kondaki K Fragiadakis GA Manios Y Assessing children's diet quality in Crete based on healthy eating index: the children study Eur J Clin Nutr 2009 63 964 969 10.1038/ejcn.2009.10 19223917

[B16] Serra-Majem L Ribas L Ngo J Ortega RM García A Pérez-Rodrigo C Aranceta J Food, youth and the Mediterranean diet in Spain. Development of KIDMED, Mediterranean diet quality index in children and adolescents Publ Health Nutr 2004 7 931 935 10.1079/phn2004556 15482620

[B17] Lazarou C Panagiotakos DB Chrysohoou C Andronikou C Matalas A-L C-Reactive protein levels are associated with adiposity and a high inflammatory foods index in mountainous Cypriot children Clin Nutr 2010 29 779 783 10.1016/j.clnu.2010.05.001 20554094

[B18] Lazarou C Panagiotakos DB Matalas A-L Foods E-KINDEX: A dietary index associated with reduced blood pressure levels among young children: The CYKIDS study J Am Diet Assoc 2009 109 1070 1075 10.1016/j.jada.2009.03.010 19465190

[B19] Kersting M Alexy U Clausen K Using the concept of food based dietary guidelines to develop an Optimized Mixed Diet (OMD) for German children and adolescents J Pediatr Gastroenterol Nutr 2005 40 301 308 10.1097/01.MPG.0000153887.19429.70 15735483

[B20] Drescher LS Thiele S Mensink GBM A new index to measure healthy food diversity better reflects a healthy diet than traditional measures J Nutr 2007 137 647 651 17311954 10.1093/jn/137.3.647

[B21] Kleiser C Mensink GBM Scheidt-Nave C Kurth BM HuSKY: a healthy nutrition score based on food intake of children and adolescents in Germany Br J Nutr 2009 102 610 618 10.1017/S0007114509222689 19203423

[B22] Kleiser C Mensink GBM Kurth BM Neuhauser H Schenk L Ernährungsverhalten von Kindern und Jugendlichen mit Migrationshintergrund - KiGGS-Migrantenauswertung - Endbericht. (Eating habits of children and adolescents with migration background - KiGGS migrants report - final report) 2007

[B23] Kamtsiuris P Lange M Schaffrath Rosario A Der Kinder- und Jugendgesundheitssurvey (KiGGS): Stichprobendesign, response und nonresponse-analyse. (The National Health Interview and Examination Survey for Children and Adolescents (KiGGS): Sample design, response and nonresponse analysis) Bundesgesundheitsblatt Gesundheitsforschung Gesundheitsschutz 2007 50 547 556 10.1007/s00103-007-0215-9 17514438

[B24] Kurth BM Kamtsiuris P Holling H Schlaud M Dolle R Ellert U Kahl H Knopf H Lange M Mensink GBM Der Kinder- und Jugendgesundheitssurvey (KiGGS): Ein Überblick über Planung, Durchführung und Ergebnisse unter Berücksichtigung von Aspekten eines Qualitätsmanagements. (The German Health Interview and Examination Survey for Children and Adolescents (KiGGS): an overview of planning, implementation and results taking into account aspects of quality management) BMC Public Health 2008 8 196 10.1186/1471-2458-8-196 17514437

[B25] Schaffrath Rosario A Kurth BM Stolzenberg H Ellert U Neuhauser H Body mass index percentiles for children and adolescents in Germany based on a nationally representative sample (KiGGS 2003–2006) Eur J Clin Nutr 2010 64 341 349 10.1038/ejcn.2010.8 20179728

[B26] Thierfelder W Dortschy R Hintzpeter B Kahl H Scheidt-Nave C Biochemical measures in the German Health Interview and Examination Survey for Children and Adolescents (KiGGS) LaboratoriumsMedizin 2008 32 10.1007/s00103-007-0238-2 17514461

[B27] Mensink GBM Burger M Was isst du? Ein Verzehrshäufigkeitsfragebogen für Kinder und Jugendliche. (What do you eat? Food frequency questionnaire for children and adolescents) Bundesgesundheitsblatt Gesundheitsforschung Gesundheitsschutz 2004 47 219 226 10.1007/s00103-003-0794-z 15205789

[B28] Knopf H Arzneimittelanwendung bei Kindern und Jugendlichen. (Medication use in children and adolescents.) Bundesgesundheitsblatt Gesundheitsforschung Gesundheitsschutz 2007 50 863 870 10.1007/s00103-007-0249-z 17514472

[B29] Winkler J Stolzenberg H Der Sozialschichtindex im Bundes-Gesundheitssurvey. (Social Status Scaling in the German national Health Interview and Examination Survey) Gesundheitswesen 1999 61 178 183 10726418

[B30] Truthmann J Mensink GBM Richter A Relative validation of the KiGGS food frequency questionnaire among adolescents in Germany Nutr J 2011 10 133 10.1186/1475-2891-10-133 22152115 PMC3261099

[B31] Berry CH Corporate growth and diversification J Law Econ 1971 14 371 383 10.1086/466714

[B32] Vollwertig essen und trinken nach den 10 Regeln der DGE. (10 rules of the DGE for wholesome eating and drinking) http://www.dge.de/pdf/10-Regeln-der-DGE.pdf

[B33] Kurth BM Lange C Kamtsiuris P Hölling H Gesundheitsmonitoring am Robert Koch-Institut Bundesgesundheitsblatt Gesundheitsforschung Gesundheitsschutz 2009 52 557 570 10.1007/s00103-009-0843-3 19343279

[B34] Rabenberg M Mensink GBM Obst- und Gemüsekonsum heute GBE kompakt 2011 2 6

[B35] Kromeyer-Hauschild K Wabitsch M Kunze D Geller F Geiß HC Hesse V von Hippel A Jaeger U Korte W Menner H Perzentile für den Body-Mass-Index für das Kindes- und Jugendalter unter Heranziehung verschiedener deutscher Stichproben. (Percentiles of body mass index in children and adolescents evaluated from different regional German studies) Monatsschr Kinderheilkd 2001 8 807 818

[B36] Bland JM Altman DG Statistics notes: Cronbach's alpha BMJ 1997 314 572 10.1136/bmj.314.7080.572 9055718 PMC2126061

[B37] Shannon J Kristal AR Curry SJ Beresford SA Application of a behavioral approach to measuring dietary change: the fat- and fiber-related diet behavior questionnaire Cancer Epidemiol Biomarkers Prev 1997 6 355 361 9149896

[B38] Fink A Ahrens W, Pigeot I Epidemiological Field Work in Population-Based Studies Handbook of Epidemiology 2007 Berlin: Springer-Verlag

[B39] Institute of Medicine Dietary Reference Intakes for Thiamin, Riboflavin, Vitamin B-6, Folate, Vitamin B-12, Pantothenic Acid, Biotin, and Choline 1998 Washington, DC: National Academy Press 23193625

[B40] Institute of Medicine Dietary Reference Intakes for Vitamin A, Vitamin K, Arsenic, Boron, Chromium, Copper, Iodine, Iron, Molybdenum, Nickel, Silicon, Vanadium, and Zinc 2001 Washington, DC: National Academy Press 25057538

[B41] Bässler K-H Grühn E Loew D Pietrzik K Vitamin-Lexikon für Ärzte, Apotheker und Ernährungswissenschaftler. (Vitamin Encyclopedia for doctors, pharmacists and nutritionists) 2002 3 München: Urban & Fischer

[B42] Bundesinstitut für Risikobewertung (BfR) Studie zu Fleischverzehr und Sterblichkeit. Stellungnahme Nr. 023/2009 des BfR vom 29. Mai 2009. (Study on meat intake and mortality. Statement no. 023/2009 of BfR on May, 29 2009) 2009

[B43] Kushi L Lenart E Willett W Health implications of Mediterranean diets in light of contemporary knowledge. 2. Meat, wine, fats, and oils Am J Clin Nutr 1995 61 1416S 1427S 7754997 10.1093/ajcn/61.6.1407S

[B44] Inzucchi SE Diagnosis of diabetes New Engl J Med 2012 367 542 550 10.1056/NEJMcp1103643 22873534

[B45] Salmerón J Manson JE Stampfer MJ Colditz GA Wing AL Willett WC Dietary fiber, glycemic load, and risk of non—insulin-dependent diabetes mellitus in women JAMA: J Am Med Assoc 1997 277 472 477 10.1001/jama.1997.03540300040031 9020271

[B46] Bach-Faig A Geleva D Carrasco J Ribas-Barba L Serra-Majem L Evaluating associations between Mediterranean diet adherence indexes and biomarkers of diet and disease Public Health Nutr 2006 9 1110 1117 17378949 10.1017/S1368980007668499

[B47] Hann CS Rock CL King I Drewnowski A Validation of the healthy eating index with use of plasma biomarkers in a clinical sample of women Am J Clin Nutr 2001 74 479 486 11566646 10.1093/ajcn/74.4.479

[B48] Weinstein SJ Vogt TM Gerrior SA Healthy eating index scores are associated with blood nutrient concentrations in the third National Health And Nutrition Examination Survey J Am Diet Assoc 2004 104 576 584 10.1016/j.jada.2004.01.005 15054343

[B49] Vaccarino V Krumholz HM Risk factors for cardiovascular disease: one down, many more to evaluate Ann Intern Med 1999 131 62 63 10391817 10.7326/0003-4819-131-1-199907060-00012

[B50] Steinberger J Daniels SR Eckel RH Hayman L Lustig RH McCrindle B Mietus-Snyder ML Progress and challenges in metabolic syndrome in children and adolescents Circulation 2009 119 628 647 10.1161/CIRCULATIONAHA.108.191394 19139390

[B51] Hunter D Willett WC Biochemical Indicators of Dietary Intake Nutritional Epidemiology 1998 New York: Oxford University Press 174 243

[B52] McNulty H Pentieva K Hoey L Ward M Homocysteine, B-vitamins and CVD Proc Nutr Soc 2008 67 232 237 10.1017/S0029665108007076 18412997

[B53] Centritto F Iacoviello L di Giuseppe R De Curtis A Costanzo S Zito F Grioni S Sieri S Donati MB de Gaetano G Di Castelnuovo A Dietary patterns, cardiovascular risk factors and C-reactive protein in a healthy Italian population Nutr Metab Cardiovasc Dis 2009 19 697 706 10.1016/j.numecd.2008.11.009 19303267

[B54] Hidaka H Takiwaki M Yamashita M Kawasaki K Sugano M Honda T Consumption of nonfat milk results in a less atherogenic lipoprotein profile: a pilot study Ann Nutr Metab 2012 61 111 116 10.1159/000339261 22907079

[B55] Hoebeeck LI Rietzschel ER Langlois M De Buyzere M De Bacquer D De Backer G Maes L Gillebert T Huybrechts I The relationship between diet and subclinical atherosclerosis: results from the Asklepios Study Eur J Clin Nutr 2011 65 606 613 10.1038/ejcn.2010.286 21245883

[B56] Drewnowski A Fiddler EC Dauchet L Galan P Hercberg S Diet quality measures and cardiovascular risk factors in France: applying the healthy eating index to the SU.VI. MAX Study J Am Coll Nutr 2009 28 22 29 19571156 10.1080/07315724.2009.10719757

[B57] McCullough ML Feskanich D Stampfer MJ Giovannucci EL Rimm EB Hu FB Spiegelman D Hunter DJ Colditz GA Willett WC Diet quality and major chronic disease risk in men and women: moving toward improved dietary guidance Am J Clin Nutr 2002 76 1261 1271 12450892 10.1093/ajcn/76.6.1261

[B58] Livingstone MB Robson PJ Measurement of dietary intake in children Proc Nutr Soc 2000 59 279 293 10.1017/S0029665100000318 10946797

[B59] Kahl H Schaffrath Rosario A Schlaud M Sexual maturation of children and adolescents in Germany. Results of the German Health Interview and Examination Survey for Children and Adolescents (KiGGS) Bundesgesundheitsblatt Gesundheitsforschung Gesundheitsschutz 2007 50 677 685 10.1007/s00103-007-0229-3 17514452

[B60] LaRosa JC Lipids and cardiovascular disease: Do the findings and therapy apply equally to men and women? Womens Health Issues 1992 2 102 113 10.1016/S1049-3867(05)80278-6 1617306

[B61] Willett WC Willett WC Overview of Nutritional Epidemiology Nutritional Epidemiology 1998 New York: Oxford University Press 3 17

[B62] Togo P Osler M Sorensen TI Heitmann BL Food intake patterns and body mass index in observational studies Int J Obes Relat Metab Disord 2001 25 1741 1751 10.1038/sj.ijo.0801819 11781753

[B63] Hedrick VE Savla J Comber DL Flack KD Estabrooks PA Nsiah-Kumi PA Ortmeier S Davy BM Development of a brief questionnaire to assess habitual beverage intake (BEVQ-15): sugar-sweetened beverages and total beverage energy intake J Acad Nutr Diet 2012 112 840 849 10.1016/j.jand.2012.01.023 22709811 PMC3379009

[B64] George CG Milani TJ Hanss-Nuss H Kim M Freeland-Graves JH Development and validation of a semi-quantitative food frequency questionnaire for young adult women in the southwestern United States Nutr Res 2004 24 29 43 10.1016/j.nutres.2003.09.006

[B65] Kristal AR Peters U Potter JD Is it time to abandon the food frequency questionnaire? Cancer Epidemiol Biomarkers Prev 2005 14 2826 2828 10.1158/1055-9965.EPI-12-ED1 16364996

[B66] Kant AK Graubard BI A comparison of three dietary pattern indexes for predicting biomarkers of diet and disease J Am Coll Nutr 2005 24 294 303 16093407 10.1080/07315724.2005.10719477

